# Cancer-associated fibroblasts in acute leukemia

**DOI:** 10.3389/fonc.2022.1022979

**Published:** 2022-12-19

**Authors:** Ling Gu, Ping Liao, Hanmin Liu

**Affiliations:** ^1^ Department of Pediatrics, Key Laboratory of Birth Defects and Related Diseases of Women and Children (Sichuan University), Ministry of Education, West China Second University Hospital, Sichuan University, Chengdu, China; ^2^ The Joint Laboratory for Lung Development and Related Diseases of West China Second University Hospital, Sichuan University and School of Life Sciences of Fudan University, West China Institute of Women and Children’s Health, West China Second University Hospital, Sichuan University, Chengdu, China; ^3^ NHC Key Laboratory of Chronobiology, Sichuan University, Chengdu, China; ^4^ Calcium Signalling Laboratory, National Neuroscience Institute, Singapore, Singapore; ^5^ Academic & Clinical Development, Duke-NUS Medical School, Singapore, Singapore; ^6^ Health and Social Sciences, Singapore Institute of Technology, Singapore, Singapore; ^7^ Sichuan Birth Defects Clinical Research Center, West China Second University Hospital, Sichuan University, Chengdu, China

**Keywords:** bone marrow, tumor microenvironment, leukemia, relapsed/refractory, cancer associated fibroblasts

## Abstract

Although the prognosis for acute leukemia has greatly improved, treatment of relapsed/refractory acute leukemia (R/R AL) remains challenging. Recently, increasing evidence indicates that the bone marrow microenvironment (BMM) plays a crucial role in leukemogenesis and therapeutic resistance; therefore, BMM-targeted strategies should be a potent protocol for treating R/R AL. The targeting of cancer-associated fibroblasts (CAFs) in solid tumors has received much attention and has achieved some progress, as CAFs might act as an organizer in the tumor microenvironment. Additionally, over the last 10 years, attention has been drawn to the role of CAFs in the BMM. In spite of certain successes in preclinical and clinical studies, the heterogeneity and plasticity of CAFs mean targeting them is a big challenge. Herein, we review the heterogeneity and roles of CAFs in the BMM and highlight the challenges and opportunities associated with acute leukemia therapies that involve the targeting of CAFs.

## 1 Background

Acute leukemia is a clonal hematopoietic cancer originating in the bone marrow (BM) and can be classified into two types: acute lymphoid leukemia (ALL) and acute myeloid leukemia (AML). With the advancement of therapies, leukemia is no longer an incurable disease. In children, the 5-year event-free survival (EFS) rate is approximately 85-90% for ALL, sometimes exceeding 90% in ALL trials in developed countries (1–4), and approximately 45-65% for AML (5, 6). However, in adults, the 5-year EFS rate for ALL is only 35-45% (7, 8). The prognosis for adult AML is better in acute promyelocytic leukemia (APL), with a 5-year EFS rate exceeding 80% (9, 10). However, only 35-40% of patients with AML manage to survive for more than 5 years (10, 11). Even in the most curable pediatric ALL, 10-15% of patients do not survive because of chemo-resistance and relapse, which is named relapsed/refractory (R/R) ALL (12, 13). The proportion of R/R AML and R/R adult ALL cases is far higher than that of R/R pediatric ALL.

Until now, the treatment of leukemia has been focused on targeting leukemic cells. While the intensity of chemoradiotherapy is limited by toxic side effects, such as pancytopenia, BM transplantation (BMT) has been drawn into the therapeutic protocol to help reconstruct hematologic and immunologic capacity following high-intensity chemotherapy and radiation treatment to eradicate leukemic cells (14–16). Even so, the prognosis for patients with R/R leukemia remains poor. The exploration of innovative approaches is crucial for patients with R/R leukemia. Immunotherapy, especially chimeric antigen receptor T (CAR-T) cell and antibody therapy, improves the response rate in patients with R/R leukemia by targeting leukemic cells (1, 8, 13–19). However, the cure rate has not been noticeably improved, especially in patients with R/R AML, which highlights an urgent need for novel and synergistic therapies.

‘Seed-and-soil’ theory is well known in cancer research and the term was coined by Dr. Stephen Paget in 1889 (20, 21). ‘Seed’ and ‘soil’ crosstalk may push cancer progression. Remodeling of the ‘soil’ will make it more difficult for cancer cells but more suitable for normal cells, thus potentially helping to cure cancer. As a matter of course, the next target should be the ‘soil’. ‘Soil’ remodeling is important for R/R leukemia patients as it may provide conditions in which cancer cells and cancer stem cells struggle to survive in (22–25). It generally accepted that the ‘soil’ of solid cancer, known as the tumor microenvironment (TME), is a target-rich environment (26–31). Cancer-associated fibroblasts (CAFs), the major players in the TME, have drawn much attention for their multiple functions, including extracellular matrix (ECM) remodeling, growth factor, cytokine, and chemokine production, angiogenesis regulation, and metabolism and immune system modulation (24, 32–39). In this review, we summarize the role of CAFs in acute leukemia and highlight the challenges and opportunities associated with CAF-targeting therapy.

## 2 Bone marrow microenvironment and CAFs

First, we must understand the ‘soil’ of leukemic cells and stem cells, the BM microenvironment (BMM). BMM plays a key role in regulating normal hematopoiesis, as well as chondrogenesis and osteogenesis. Initially, BMM was identified as necessary for successful BMT to reconstruct hematopoiesis. In the 1950s, few patients with leukemia benefitted from BMT (40). After the human histocompatibility antigen system was recognized, a modern era of human BMT began. From then on, the BMM has been slowly demystified.

In 1961, Fliedner et al. (41) pointed out that the recovery of hematopoiesis in rats following 1000 cGy total body irradiation required the recovery of vasculogenesis as support. Then, in 1967, Wolf and Trentin applied the term ‘hemopoietic inductive microenvironment’ to this event in the spleen and BM (42–44). In 1978, Raymond Schofield (45) formally proposed the ‘stem cell niche’ in BM as a specialized microenvironment for stem cells *in vivo*. Since the 1980s, an increasing number of studies have showed that the BM niche (also called BMM) plays a crucial role in both hematopoiesis and leukemogenesis (46–51). Traditionally, the BMM was divided into endosteal and vascular niches, which may participate in different divisions of labor (52–61). Through technological breakthroughs, such as the construction of transgenic mouse models, the development of sophisticated imaging technologies, and single-cell sequencing, the atlas of BMM is becoming clearer. BMM is a continuum in which hematopoietic stem cells (HSCs) and leukemic stem cells (LSCs) may locate in their corresponding niche. The trouble is that LSCs remodel the BMM into a leukemia-permissive microenvironment while suppressing a hematopoietic-permissive microenvironment (50, 60, 62–67). Clinically, this hypothesis is best supported by donor cell leukemia, in which leukemia originates from engrafted donor cells after allogeneic HSC transplantation, i.e., the leukemia-permissive microenvironment may initiate leukemogenesis in healthy cells (68–72). Therefore, targeting of the leukemia-permissive BMM to restore hematopoietic-permissive BMM can be a useful strategy for overcoming R/R leukemia. Herein, the next issue is to dig out the potent target cells.

### 2.1 The cell components of BMM

Initially, in the 1960s, Owen and Macapheson (73, 74) observed a group of pre-osteoblasts growing in the inner periosteal surface of the femur. In 1968, Friedenstein (75) and Tavassoli et al. (76) found that BMT could generate non-hematopoietic osteogenic cells. Then, in the 1980s, many papers reported fibroblast colonies originating from stromal osteogenic precursor cells in BM (77–81). In 1991, Caplan (82) termed precursor cells with multipotency properties as mesenchymal stem cells (MSCs). In the present day, autoradiography, BM smear and biopsy, flowcytometry, *in vivo* BMT, and *in vitro* cell culture have helped us recognize the cellular components of the BMM, including MSCs, endothelial cells (ECs), adipocytes, Cxcl12-abundant reticular (CAR) cells, osteogenic cells, macrophages, fibroblasts, Schwann cells, and possibly other stromal cells (81, 83, 84). Cre-mediated lineage tracing and deletion of molecular factors helped trace cell fate and differentiation, which were still limited in a small piece of a whole. Recently, single-cell and spatial transcriptomic technologies provided the first systematic and label-free identification of cell types of the BMM (85–90). So far, we can map the cellular composition and distribution in the BMM. Different BM resident cell types are successfully allocated to endosteal, sinusoidal, arteriolar, and non-vascular niches (90). Baryawno et al. (86) first profiled all non-hematopoietic (Ter119-/CD71-/Lin-) cells in mouse BM and gained 17 clusters spanning MSCs (*Lepr^+^Cxcl12^+^*), osteolineage cells (*Bglap^+^*), chondrocytes (*Acan^+^Col2a1^+^*), fibroblasts (*S100a4^+^*), BMECs (*Cdh5^+^*), pericytes (*Acta2^+^*), and possible transitional states. Based on single-cell and spatial transcriptomics, Baccin et al. (87), identified nine cell types in BM-resident non-hematopoietic cells and demonstrated their differential localization, including two different EC clusters (*Ly6a* ^+^ arterial ECs and *Emcn* ^+^ sinusoidal ECs), CAR cells (*Lepr^high^* Adipo-CAR and *osterix^high^ Lepr^low^* Osteo-CAR), three distinct fibroblast clusters (stromal, arteriolar, and endosteal localizations), myofibroblasts, Ng2^+^ Nestin^+^ MSCs, chondrocytes (*Acan* and *Sox9*), osteoblasts (*Osteocalcin*/*Bglap* and *Col1a1*), smooth muscle cells (*Tagln* and *Acta2*), and Schwann cells (*Mog*, *Mag*).

In the BMM, MSCs and ECs are the most abundant subsets (86), and have been fully researched, especially MSCs. Fibroblasts, myofibroblasts, and Schwann cells were found to be more abundant in crushed bones than in flushed bones (87). Therefore, these cells might be ignored during regular clinical examinations without broken bones, such as BM aspiration and biopsy, due to the limited number of cells. Baryawno et al. (86) revealed that Fibroblast-1 and -2 cells are MSC-like as they expressed the progenitor marker CD34 and MSC markers (*Ly6a*, *Pdgfra*, *Thy1*, *and Cd44*), but not BMECs or pericytes genes (*Cdh5* and *Acta2*). While in the BMM of AML, *Cxcl12*, *Kitl*, and *Angpt1* were upregulated in Fibroblast-1 cells (similar to Cxcl12-secreting CAFs). CAFs are defined as fibroblasts that are located within or adjacent to cancer cells, and have been extensively studied due to the ease with which they can be obtained and cultured *in vitro* from solid cancers (24, 33, 35, 91). In the past decade, CAFs have been well recognized as a promising target in the TME (25, 33, 34, 37).

### 2.2 Origins of CAFs in BM

Fibroblasts are defined as interstitial cells of a mesenchymal lineage that are not epithelial, endothelial, or immune cells (34, 37, 92). The origins and roles of fibroblasts in different tissues remain ambiguous, resulting in a lack of unified biomarkers to define them (36, 93). It is generally accepted that CAFs are the main participants in ECM remodeling, wound-healing responses, immune cell recruitment, inflammation, and fibrosis (32, 34–37, 93). The origins and roles of CAFs are even more complicated than fibroblasts. So far, over 10 origins of CAFs have been found in solid tumors, including tissue-resident cells (fibroblasts, myofibroblasts, fibrocytes, epithelial cells, endothelial cells, adipocytes, smooth muscle cells, and immune cells) and BM-derived cells (MSCs, circulating fibrocytes, and immune cells) (24, 35, 37). Still, the precise origins of CAFs and CAF subgroups, and the differences between CAFs and fibroblasts in normal tissues, remain elusive due to the phenotypic and functional plasticity of these cells and the lack of well-defined lineage biomarkers (34, 37). However, based on scRNA-seq and spatial transcription technology, there is a considerable understanding of the heterogeneity of CAFs in solid cancers, such as pancreatic cancer, liver cancer, gastric cancer, head and neck cancer, and breast cancer (94–102).

**Figure 1:**
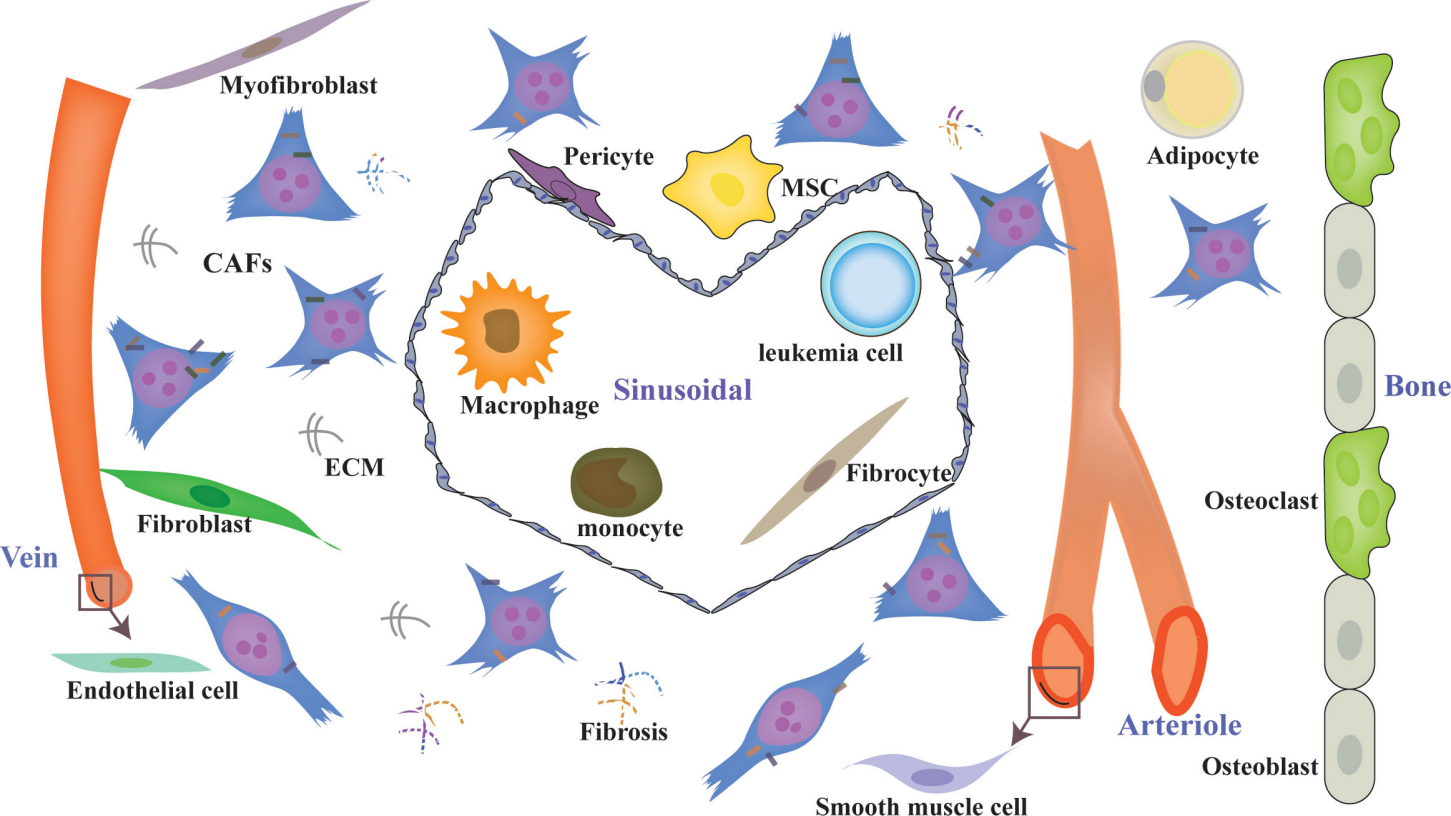
Diverse origins of CAFs in the BMM. CAFs can originate from diverse sources, such as MSCs, fibroblasts, myofibroblasts, fibrocytes, smooth muscle cells, endothelial cells, adipocyte pericytes, monocytes, macrophages, and leukemia cells, with different phenotypes. CAFs are a heterogeneous population with distinct functions in the BMM. ECM, extracellular matrix; CAFs, cancer-associated fibroblasts.

**Figure 2:**
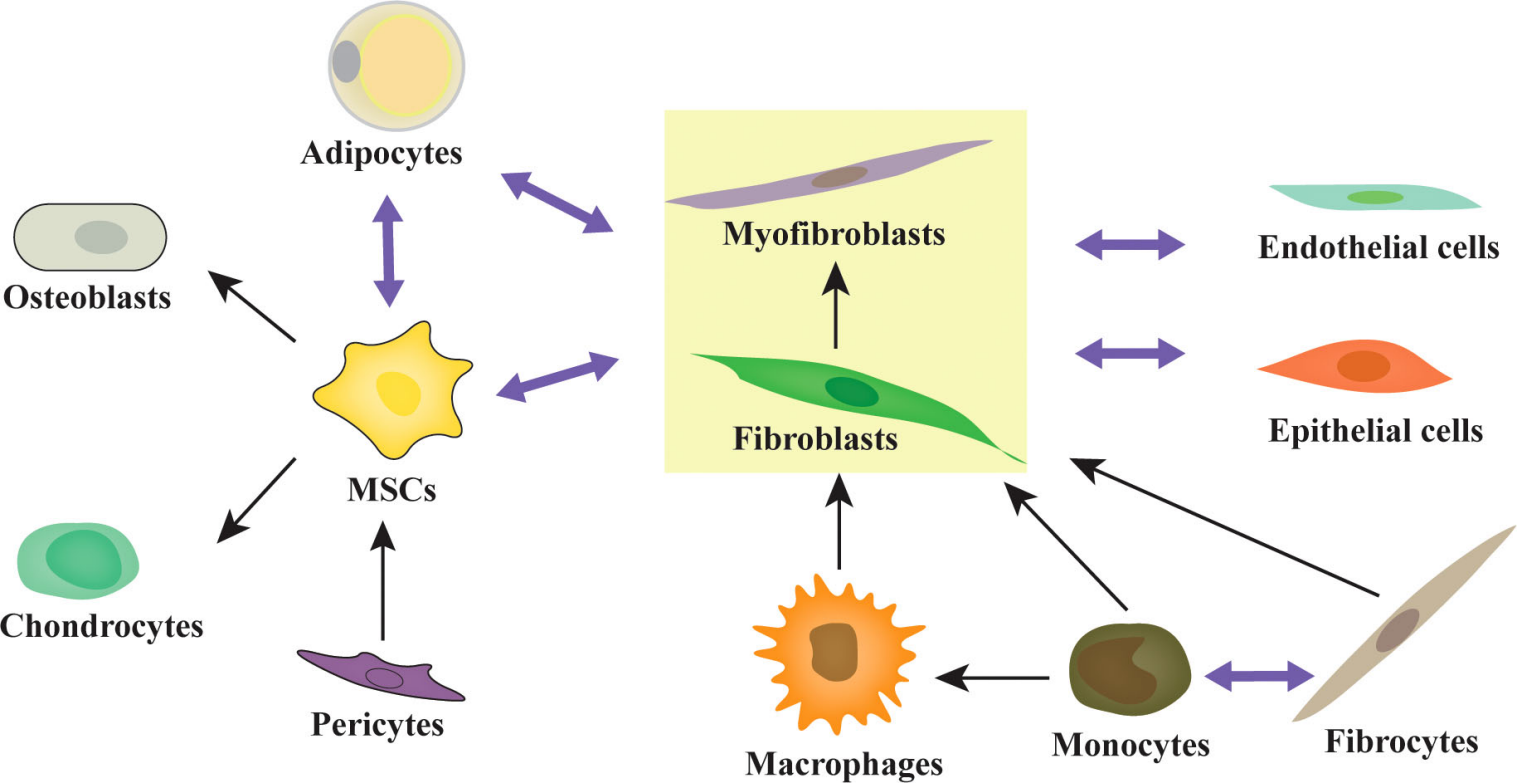
The multipotent differentiation capacity of origin cells of CAFs. Activated fibroblasts, MSCs, and adipocytes are highly plastic and exhibit multipotent capacity. MSCs can differentiate into osteoblasts, chondrocytes, adipocytes, and fibroblasts/myofibroblasts. Adipocytes can differentiate into fibroblasts/myofibroblasts and osteoblasts. Fibrocytes can differentiate into monocytes, fibroblasts/myofibroblasts, adipocytes, osteoblasts, and chondrocytes. Monocytes can differentiate into fibrocytes, macrophages, and fibroblasts/myofibroblasts. The pericytes and adipocytes can differentiate into MSCs. Similarly, activated fibroblasts can transform into MSCs, adipocytes, chondrocytes, endothelial cells, ECs, and pericytes.

Although the BMM has been studied extensively since 1978, research on CAFs in hematological malignancies is falling far behind that of solid tumors. The major reason for this is that BM biopsy specimens are relatively hard to obtain. Additionally, lineage tracing of CAFs might be more difficult in BM. According to the achievements with solid tumors, we can conclude that there are abundant resident origins of CAFs in the BMM, such as MSCs, fibroblasts, myofibroblasts, fibrocytes, smooth muscle cells, endothelial-mesenchymal transformation cells, adipocyte-mesenchymal transition cells (24, 35, 103), pericyte-fibroblast transformation cells (104, 105), monocyte-fibroblast transition cells (106, 107), macrophage-mesenchymal transformation cells (108), and leukemia cells (109–111) (**Figure 1**). Different cell origins of CAFs might suggest different phenotypes and roles. Additionally, most of the cell origins of CAFs in BM contain populations with multipotent differentiation capacity, which may make lineage tracing of CAFs more difficult (**Figure 2**). For example, MSCs can differentiate into osteoblasts, chondrocytes, and adipocytes *in vitro* and *in vivo* (82, 112, 113). Adipocytes can differentiate into myofibroblasts (103) and osteoblasts (114). CD34(^+^) fibrocytes are BM-derived monocyte progenitor cells, which can differentiate into adipocytes, osteoblasts, and chondrocytes (115, 116). Monocytes can differentiate into fibrocytes and macrophages (117, 118). Furthermore, the cell origins of BM MSCs currently remain unclear; a mesodermal, a neuro-ectodermal, or even a dual origin have been suggested (113). The pericytes of ectodermal origin can differentiate into MSCs (113, 119, 120). BM MSCs may arise from BM or adipose tissue (121). Similarly, activated fibroblasts can transform into MSCs, adipocytes, chondrocytes, endothelial cells, ECs, and pericytes, and can even be induced to become induced pluripotent stem cells (iPSCs) (35, 122).

## 3 CAFs in the BMM of leukemia

### 3.1 Myelofibrosis and CAFs

First described in 1879, BM fibrosis with fibroblast infiltration and excessive ECM deposition (123, 124) is a typical type of BMM remodeling (125). Now, myelofibrosis (MF) is defined as a clonal hematopoietic BCR-ABL-negative myeloproliferative neoplasm characterized by BM fibrosis, extramedullary hematopoiesis, megakaryocytic hyperplasia, and constitutional symptoms (126). MF may be primary or secondary with a heterogeneous clinical course, ranging from a chronic asymptomatic state to acute leukemic transformation, and possibly a preleukemic state (126, 127). Most forms of secondary MF collaborate with myeloproliferative neoplasms (MPN) and chronic myeloid leukemia (CML). Leukemia transformation is rare in patients with non-fibrotic MPNs but common in patients with MF (128–131). Patients with acute leukemia transformed from MF have a dismal prognosis, with a median survival time of approximately 3 months (128, 129). BM fibrosis in ALL and AML was first described in 1964 (132). Although BM fibrosis may disappear and accompany the complete remission of leukemia, a higher degree of fibrosis (measured as reticulin fibrin density) may correlate with relapse and higher minimal residual disease (MRD) in ALL, especially B-ALL (133–136), and with a poor prognosis in AML (137); however, there remains controversy (138, 139). These results imply that CAFs may play a crucial role in a part of patients with acute leukemia.

### 3.2 Acute leukemia and CAFs

The phenotypes and roles of BM CAFs were first reported in patients and mice with multiple myeloma (MM) in 2014 (140). The same year, Duan et al. (141) found that ALL cells may induce a dynamically transient niche in the BMM with the help of chemotherapy: beginning with Nestin^+^ MSCs, maturating through their transition to a-SMA^+^ cells, and terminating with fiber residues, called the NSM niche, in mice models and patients with ALL after chemotherapy. The NSM niche was associated with additional difficulties in achieving complete remission after therapy in ALL patients, i.e., the transit of Nestin^+^ MSCs to a-SMA^+^ CAFs might correlate with BM fibrosis and poor prognosis in ALL. In 2015, Paggetti et al. (142) reported that exosomes from chronic lymphoid leukemia (CLL) may induce the transition of BM MSCs to CAFs. In 2016, a retrospective study on BM biopsies from patients with AML showed that CAFs were widespread within the BM. Furthermore, excessive reticular fibers in the BM led to a higher frequency of relapse and mortality in primary ALL patients (143). In 2019, Burt et al. (144) pinpointed that CAFs/activated MSCs are frequently presence in ALL, which could prevent ALL cell apoptosis and death from reactive oxygen species-inducing agents by mitochondrial transfer. Exposure to Ara-C or daunorubicin may generate CAFs *in vitro* and in ALL mice models (144). Then, Pan et al. (145, 146) found that TGF-β is a key factor for BM MSCs to obtain a CAF-like phenotype in a B-ALL microenvironment, which may interact with ALL cells through an SDF1-CXCR4 signaling axis to promote the progression of B-ALL. Using single-cell sequencing, Baryawno et al. (86) revealed a decrease in Fibroblast-5s (*Sox9*, *Spp1*, *Nt5e*, *cspg4*, and *clip)*, an increase in Fibroblast-2s (*Cd34*, *Ly6a*, *Pdgfra*, *Thy1*, and *Cd44*), and a Cxcl12-secreting CAF phenotype of Fibroblast-1s (with upregulation of *Cxcl12*, *Kitl*, and *Angpt1*) in mice BM with AML. In 2021, our team established the first CAF tumor cell line, HXWMF-1 (α-SMA, vimentin, HSP47, S100A4/FSP1, FAP, PDGFRβ, and CD34 positive) (147), originated from the subcutaneous xenografts of HXEX-ALL1 (148), a cell line from a relapsed patient with B-ALL. The cell line provides firm evidence that leukemia cells may induce malignant transformation of CAFs (147). Malignant CAFs might remodel the BMM to form a more aggressive niche. Although the exact roles and underlying mechanisms of CAFs in BM remain elusive, it is clear that CAFs in BM may correlate with BM fibrosis, promote leukemia progression, and induce chemoresistance (**Table 1**) (86, 141, 143–146). Chemotherapeutic drugs, such as Ara-C and daunorubicin, may induce the generation of CAFs (**Table 1**) (141, 144). In general, CAFs in BM may have distinct phenotypes and play crucial roles in leukemogenesis and therapy resistance. Understanding the role of CAFs in BM and AL may have clinical significance as it may facilitate the identification of novel drug targets for BMM and immunotherapy.

**Table 1:** CAFs in AL and their functions in leukemia progression.

Models	Induction factors of CAFs	CAFs subtypes	Cell origin of CAFs	Roles of CAFs	Mechanisms	Method	Correlated with BM fibrosis	References
**Murine ALL models /Pediatric and Adult patients with ALL**	Ara-C or DNR	a-SMA^+^, Vimentin^+^ (murine)/a-SMA^+^, Nestin^-^, CD146^-^ (human)	MSCs with Nestin^+^	Protect leukemic cells from chemotherapy	GDF15 mediated the niche protection	IF and IHC	Yes	Duan et al. (140)
**Adult patients with AML**	NA	FSP1^+^, α-SMA^+^, or FAP^+^	MSCs	Protect leukemic cells from chemotherapy	GDF15 mediated the chemoprotection	IHC	Yes	Zhai et al. (142)
**Adult patients with ALL /Murine B-ALL models**	Ara-C or DNR	F-action^+^, α-SMA^+^	MSCs	Protect leukemic cells from chemotherapy	Mitochondria transfer mediated the chemoprotection.	Cell culture and IF	NA	Burt et al. (143)
**Murine AML models**	NA	Cd34^+^, Ly6a^+^, Pdgfra^+^, Thy1^+^ and Cd44^+^/ Cxcl12^+^, Kitl^+^, and Angpt1^+^	NA	NA	NA	Single cell sequencing	NA	Baryawno et al. (86)
**Adult patients with B-ALL**	TGF-β	α-SMA^+^ , FAP^+^	MSCs	Accelerate leukemic cells migration and invasion	NA	Cell culture, IF	NA	Pan et al. (145)
**Adult patients with B-ALL/ Murine B-ALL modle**	TGF-β	a-SMA^+^, FAP^+^	MSCs	Promoting the growth and invasion of B-ALL	SDF-1/CXCR4 axis mediated the communication of CAFs and leukemia cells	IHC	Yes	Pan et al. (144)

IF, immunofluorescence; IHC, immunohistochemistry; NA, not reported.

### 3.3 Genetic alteration and CAFs

G-banding analysis showed that HXWMF-1 cells have 60–70 chromosomes with complex structural chromosomal abnormalities (147), which raises the question of whether there are cytogenetic abnormalities in BM stromal cells in patients with acute leukemia? Some studies reported that stromal cells in the BM of MM, myelodysplastic syndrome (MDS), AML, ALL, and CML patients had numerical and structural chromosomal abnormalities, which were different from the abnormalities of leukemic cells (149–153). However, other researchers were unable to find chromosomal abnormalities in stromal cells from different hematological diseases, including MDS, AML, ALL, CLL, and CML (154–157). Gunsilius et al. (158) reported that ECs from patients with CML expressed the *BCR-ABL* fusion gene. Zhou et al. (159) found that clonal expansion of fibroblasts with somatic copy number alterations is prevalent in patients with colorectal cancer. The genetic profile of cancer cells can affect the surrounding stoma (160), and genetic alterations have been detected in a few stroma cells in solid tumors (161–163). In general, cytogenetic alterations could appear in stromal cells in some patients with leukemia but not all. The presence of chromosomal aberrations in BM MSCs has been associated with a bad prognosis (150).

## 4 Why focus on CAFs

### 4.1 The role of CAFs

Although, studies of the BMM of malignant hematological diseases have suggested a tumor-promoting role for CAFs (140–146, 164), studies on solid tumors revealed highly heterogeneous phenotypes in CAFs, with both tumor-promoting and restraining functions (35, 36, 38, 93, 165–169), which may partly explain the failure in clinical trials of targeting CAFs as a whole (34). The former phenotype represents most of the CAF population (38), which helps reprogram malignant ECM, increase angiogenesis and neovascurization, fuel cancer cells, direct cancer cell proliferation, metastasis, and invasion, deregulate metabolism, induce epigenetic reprogramming, unlock phenotypic plasticity, promote the stemness of cancer cells, resist cell death, shape the tumor immune microenvironment, and confer therapeutic resistance (32, 33, 36, 38, 39, 91, 93, 96, 97, 99, 168, 170, 171). Therefore, CAFs may participate in constructing almost all the fourteen hallmarks of cancer proposed by Dr. Hanahan and Dr. Weinberg (172–174), including acquiring capabilities for sustaining proliferative signaling, evading growth suppressors, resisting cell death, enabling replicative immortality, tumor-promoting inflammation, inducing/accessing vasculature, activating invasion and metastasis, reprogramming cellular metabolism, avoiding immune destruction, genome instability and mutation, unlocking phenotypic plasticity, non-mutational epigenetic reprogramming, polymorphic microbiomes, and senescent cells (172). Kochetkova and Samuel (33) reviewed the published evidence and summarized that CAF-mediated differentiation may give rise to cancer-associated immune cells, adipocytes, nerves, endothelia, and vasculature. They pointed out that CAFs are well equipped to assume the role of master organizer in the cancer by interacting with cancer cells and other stromal cells and immune cells in the TME, and producing cancer-specific ECM and secretome. Therefore, targeting CAFs to destroy cancer might be a potent therapeutic protocol for improving and perfecting cancer therapy.

### 4.2 Targeting CAFs and the associated challenges

The first clinical trial of targeting CAFs was reported in 1994, using iodine 131-labeled monoclonal antibody F19 (^131^I-mAbF19) to target FAP^+^CAFs in colorectal carcinoma patients with hepatic metastasis. The results prompted the diagnostic and therapeutic applications of mAbF19 (175). Then, an increasing number of preclinical and clinical trials of different targets and strategies were undertaken or are still in progress. However, in recent years, CAFs have been the focus of debate. There are numerous obstacles and challenges in targeting CAFs, such as a lack of specific CAF cell markers and signaling pathways, and the heterogeneous roles of CAFs. Increasing evidence has added further complication by indicating that the phenotypes of CAFs are dynamic and able to interconvert depending on tumor status, culture conditions, and therapeutic protocols (34, 93, 176–178). This presents a challenge and an opportunity, as modulating the phenotype of CAFs from tumor promoting to tumor restraining might be an attractive approach for cancer treatment (38, 93). Unfortunately, owing to the same difficulty, there are no definite and standardized markers to classify the functional subtypes of CAFs. Traditionally, α-smooth muscle actin (α-SMA) was identified as a marker of active CAFs and a prognostic factor in tumor patients; however, certain subtypes of CAFs are characterized by a far lower degree of α-SMA (176, 179, 180). Currently, a number of markers, such as α-SMA, FAP, PDGFRα/β, vimentin, S100A4 (FSP1), CAV1(caveolin 1), transgelin (TAGLN), periostin (POSTN), podoplanin (PDPN), integrin α11β1 (ITGA11), collagen type XI alpha I chain (COL11A1), and microfibril-associated protein 5 (MFAP5), are used to identify CAF populations and subgroups (24, 32, 38, 93, 179). Just as Dr. Song mentioned, CAFs are frequently defined by what they are not, typically using multiple biomarkers, resulting in an incomprehensive definition of a CAF (38). Recently, novel CAF-specific biomarkers were discovered in different cancers, such as CD10^+^GPR77^+^ CAFs in breast and lung cancer (25), G protein-coupled receptor 30^+^ CAFs in prostate cancer (181), netrin G1^+^ CAFs (182), neuregulin^+^ CAFs (183), leucine-rich-repeat-containing 15^+^ CAFs (91), Gli1^+^ CAFs (184), CD105^+^ CAFs in pancreatic cancer (99), and EGRhigh CAFs in adult T cell leukemia/lymphoma (185). Novel markers may help to precisely attack the tumor-promoting CAFs.

According to the target spot, there are two strategies for targeting CAFs, direct and indirect, which were recently comprehensively reviewed by Saw et al. (38). The direct targeting approach includes CAF depletion *via* cell markers, inhibition of CAF activation by targeting the signaling pathway, halting infiltration of CAFs, and reprogramming tumor-promoting CAFs to a quiescent state or tumor-restraining phenotype (24, 32, 38, 39). The indirect targeting approach includes targeting the TME, CAF-derived ECM, and downstream effectors (24, 32, 38, 39). However, parts of the clinical trials of targeting CAFs ended in failure, and in some cases, even accelerated cancer progression (34). Recently, there have been numerous studies on FAP-specific CAR-T cells, which can specifically attack FAP^+^ CAFs with concomitant antitumor efficacy and no severe toxicity (24, 186–188). CAR-T, which was first described by Gross et al. (189) in 1989, can enable T cells to recognize antigens independent of major histocompatibility complex II. The first FDA-approved CAR-T cell therapy obtained a good response in aspects of patients’ ALL (190, 191). CAR-T cell therapy is mainly performed in patients with hematological malignancies and is a revolutionary new treatment for cancer (192). However, responses are transient in patients as CAR-T cells may become exhausted/dysfunctional. Recently, Sakemura et al. (164) constructed a dual-targeting BCMA-FAP and BCMA-SLAMF CAR-T to target both malignant plasma cells and BM CAFs. The results showed that dual-targeting of CAR-T can overcome BM-CAF-mediated inhibition of BCMA-CAR-T (targeting plasma cells only) in an MM mice model. This study is a perfect preclinical attempt to target both cancer cells and the TME with immunotherapeutic strategies, and a brand-new attempt at targeting CAFs in the BMM. Encouragingly, the study suggests that FAP-CAR-T can be applied to target BM CAFs in hematologic malignancies to combat BMM-mediated therapy resistance.

## 5 Conclusions and perspectives

Studies on CAFs are exciting and critical for leukemia treatment. The challenge is to better understand the heterogeneity and plasticity of CAFs, which may help to develop novel CAF-targeting therapeutic strategies. Compared with solid tumors, the targeting of CAFs is more challenging in hematological malignancy. First, the BM biopsy samples are harder to obtain. To complicate matters further, it is difficult to obtain enough CAFs through regular BM aspiration and biopsy, whereas fibroblasts and myofibroblasts are abundant in crushed bones (87). Second, the precursor cells in the BMM are more complex and plastic, which make lineage tracing more challenging. In general, there are still many questions about CAFs in the BMM that need to be answered, including the following:

1. What kinds of CAFs in the BMM might correlate with R/R AL? What are their cell origins? Do these CAFs have chromosomal alterations?
2. Do CAFs contribute to donor cell leukemia? What kinds of CAFs might induce donor cell leukemia? What are the underlying mechanisms?
3. What kinds of ALs might induce the malignant transformation of CAFs? What are the exact roles of malignant CAFs?
4. What are the underlying mechanisms of the transition of precursor cells to CAFs? Are there any influences of therapeutic protocols on the transition of CAFs?

## Author contributions

LG drafted the manuscript and prepared the figures. LG and PL conceived the review. HL designed and revised the review. All authors contributed to the article and approved the submitted version.
